# Small bowel obstruction and perforation attributed to tubo-ovarian abscess following 'D’ and 'C’

**DOI:** 10.1186/1749-7922-8-41

**Published:** 2013-10-09

**Authors:** Elroy Patrick Weledji, Felix Elong

**Affiliations:** 1 Anatomy and Clinical Surgery, Faculty of Health Sciences, Regional Hospital Buea, University of Buea, Buea, S.W. Region, Cameroon; 2 Obstetrics and Gynaecology, Faculty of Health Sciences, Regional hospital, University of Buea, Buea, Cameroon

**Keywords:** Dilatation and curettage, Pelvic abscess, Intestinal obstruction, Perforation

## Abstract

We report the case of a young woman who was admitted because of small bowel obstruction and localized peritonitis following a dilatation and curettage ('D’ and 'C’) of uterus in abortion. As infection, like tubo-ovarian abscess may complicate any abortion, it seems wise to ensure that it does not exist prior to performing a 'D’ and 'C’.

## Introduction

Small bowel obstruction is a serious and costly medical condition indicating often emergency surgery. Currently small bowel obstruction required 948,000 hospital days annually, amounting for $3.2 billion, with a rate of 117 hospitalizations per 100,000 people. It constitutes 1.9% of all hospital and 3.5% of all emergency admissions that has led to laparotomy in the United States [1]. Tubo-ovarian abscess is often thought to arise from repeated episodes of pelvic inflammatory disease (PID) but may also arise after perforations of septic or even therapeutic abortions; after adnexial surgery or caeserian section; from a ruptured appendix; with pelvic malignancy, or rarely after apparently uncomplicated minor gynaecological procedures including removal or insertion of intra-uterine devices and deliveries [2-4]. Small bowel obstruction attributed to tubo-ovarian abscesses have been reported but without a link to a precipitating factor such as in this case- the 'D’ and 'C’ procedure [5-7].

### Case presentation

A 22-yr old woman (G2 P1011) was admitted as an emergency with a gradual onset severe colicky central abdominal pain 1 week after a termination of pregnancy at 16 weeks gestation. The pain became more frequent on a background of a constant lower abdominal pain. There was associated central abdominal distension, copious bilious vomiting following meals, absolute constipation and fever. There was no vaginal discharge. She had undergone a normal vaginal delivery 15 months previously. On examination she was in great distress, lying still but restless with each episode of colic. She was dehydrated and tachypnoeic. Her blood pressure 100/60 mmHg, heart rate 90/min and temperature 39°C. She had a distended abdomen with visible peristalsis and generalized rebound tenderness. Adnexal structures were unable to be palpated. The clinical impression was small bowel obstruction secondary to peritonitis from a perforated uterus as a complication of the 'D’ and 'C’. Her haemoglobin level was 12.2 gms/d but a white cell count was not available. An abdominal ultrasound scan from the referral clinic revealed a non-gravid uterus with dilated loops of bowel and free intraperitoneal fluid. Following resuscitation with intravenous fluids, nasogastric suction, intravenous antibiotics and analgesia she underwent a laparotomy.

Laparotomy revealed copious (_~_ 1-2l) amount of clear, 'transudate’ fluid in the peritoneal cavity associated with a markedly distended small bowel. There was a localized area of terminal ileal stricture at the site of adhesion of a right tubo-ovarian abscess of about 6 cm in diameter. Immediately proximal to the stricture was dilated small bowel with serosal tears suggesting impending perforation. There was a short segment of a distally collapsed terminal ileum. On mobilisation, a large amount of pus drained from the tubo-ovarian mass into the terminal ileum i.e. an internal tubo-ovarian small bowel fistula. Apart from an inflammatory exudate surrounding the uterus there was no perforation. The left adnexa was normal. A retroileal appendix adherent to the infundibulo-pelvic ligament appeared normal. The distended bowel was decompressed by suction via the terminal ileal perforation. The strictured segment of small bowel including the perforation was resected. A salpingo–oophorectomy and an appendicectomy were done. A manual end-to-end ileal anastomosis was fashioned and the abdominal cavity thoroughly lavaged with copious amount of saline. No drain was inserted because of the friable nature of the bowel and the localized nature of the peritonitis. Unfortunately, due to financial difficulties, microbiology of the purulent exudate was not requested and the excised specimen was not sent for histological examination. She received a therapeutic course of intravenous ceftriaxone 1 gm tds and metronidazole 500 mg tds for 7 days that covered the aerobes and anaerobes for a week. Apart for an ileus of 3 days, her recovery was uneventful. She was discharged on the 9th postoperative day on a 1 week course of doxycycline against *Chlamydia trachomatis* a frequent cause of pelvic inflammatory disease.

## Discussion

During surgical abortion, perforation of the uterus can occur or there may be damage to the cervix, which can predispose to the risk of preterm labour in subsequent pregnancies (cervical incompetence) [2]. There is also an increased risk of injury to infected tissue such as a tubo-ovarian abscess and spreading of the infection [3,4]. In general, surgical procedures of the female genital tract place the patient at risk for pelvic inflammatory disease, with about 15% of pelvic infections occurring after procedures that break the cervical mucous barrier [8]. The incidence of upper genital tract infection associated with first-trimester abortion is about 1 in 200 cases and the incidence of complications after a first-trimester D&C is 1.7% [6]. Uterine perforation with small bowel involvement is rare in 1^st^ trimester abortion. Shulman et al. [9] reported a case of uterine perforation and small bowel incarceration two days after a first trimester surgical abortion and correlated the sonographic and surgical findings. Without a preliminary ultrasound scan it is uncertain in this case if the tubo-ovarian abscess was present at the time of the 'D’ and 'C’ or was a complication of the procedure. The clinical course of the combined complications of a tubo-ovarian abscess with small bowel obstruction and small bowel perforation can be explained in four possible patterns: (1) contaminated curettage instruments, (2) pre-existing tubo-ovarian abscess, (3) 'sealed –off’ tubo-ovarian perforation and (4) unrecognized uterine injury with intra-abdominal involvement [9-12]. The evidence for (2) and (3) is that pelvic inflammatory disease may fix the uterus and moving it with dilators may tear it, spread the pus, and cause a fatal peritonitis [3]. The evidence for (4) is the presence of the ileal perforation within the abscess cavity and, the rarity of the reverse occurring - a tubo-ovarian abscess perforating into small bowel [5,13]. In a clinically stable patient diagnosed with small bowel obstruction associated with pelvic inflammatory disease, conservative treatment including antibiotic therapy should be attempted before any operative intervention is considered [6]. Although laparoscopic adhesiolysis requires a specific skill set and may not be appropriate in all patients it demonstrates a benefit in 30-day morbidity and mortality but should be performed by experienced laparaoscopic surgeons [14,15]. Laparoscopic management of acute peritonitis is also well established [16] Table 1.

**Table 1:** Published articles on bowel obstruction due to tubo-ovarian abscess

**Authors and year of publication**	**Country**
Weledji et al., 2013	Cameroon
Pines et al., 2008	Israel
Harel et al., 2003	USA
Malcolm, 1915	UK

## Conclusion

This case highlights the importance of requesting an ultrasound scan of the pelvis prior to performing a dilatation and curettage for abortion. This would not only confirm an intrauterine pregnancy but may also reveal an ectopic pregnancy, a co-existing tubo-ovarian abscess or other adnexal pathology.

## Consent

“Written informed consent was obtained from the patient for publication of this Case report and any accompanying images. A copy of the written consent is available for review by the Editor-in-Chief of this journal” 13. 07; 41473.

## Competing interests

Both authors declare that they have no competing interests.

## Authors’ contributions

EPW is the main author and surgeon; FE rendered advise an did some literature search. Both authors read and approved the final manuscript.
